# Steroid hormones in hair reveal sexual maturity and competition in wild house mice (*Mus musculus domesticus*)

**DOI:** 10.1038/s41598-019-53362-4

**Published:** 2019-11-15

**Authors:** Esther H. D. Carlitz, Jan-Niklas Runge, Barbara König, Lennart Winkler, Clemens Kirschbaum, Wei Gao, Anna K. Lindholm

**Affiliations:** 1 Department of Psychology, Biological Psychology, Technical University of Dresden, Dresden, Germany; 2 Department of Evolutionary Biology and Environmental Studies, University of Zurich, Zürich, Switzerland; 3 Department of Applied Zoology, Technical University of Dresden, Dresden, Germany

**Keywords:** Ecology, Community ecology

## Abstract

Endocrine data from wild populations provide important insight into social systems. However, obtaining samples for traditional methods involves capture and restraint of animals, and/or pain, which can influence the animal’s stress level, and thereby undesirable release of hormones. Here, we measured corticosterone, testosterone and progesterone in the hair of 482 wild-derived house mice that experienced sexual competition while living under semi-natural conditions. We tested whether sex, age, weight and indicators of sexual maturity, reproduction and social conflicts predict hormone concentrations measured in hair (sampling at endpoint). We show that body weight, sex and age significantly predict cumulative testosterone and progesterone levels, allowing the differentiation between subadults and adults in both sexes. Corticosterone was only slightly elevated in older males compared to older females and increased with the level of visible injuries or scars. Testosterone in males positively correlated with body weight, age, testes size, and sperm number. Progesterone in females significantly increased with age, body weight, and the number of embryos implanted throughout life, but not with the number of litters when controlled for age and weight. Our results highlight the biological validity of hair steroid measurements and provide important insight into reproductive competition in wild house mice.

## Introduction

Understanding the environmental control and evolutionary significance of the interplay between hormones and behaviour is a challenge in the fields of behaviour and physiology. A crucial aspect is being able to measure and interpret hormone levels under natural conditions. Natural conditions imply for sexually reproducing species the possibility of reproductive competition, in which males may compete with each other for access to females^1^, and in which females may compete with each other for access to resources necessary to rear young or for access to mates^2,3^. Hormone level measurements have been important in interpreting such competition, particularly between males^4,5^.

Measurements of hormones have traditionally involved saliva, blood, urine and faecal sampling. These have proved highly valuable in assessing current or recent hormone levels. However, there are limitations to these methods. Obtaining the samples often involves capture and restraint of an animal, and/or pain, which can influence the animal’s stress level, and thereby the release of hormones. Sampling hair instead may solve some of these problems, as it is little or non-invasive, and hormone concentrations measured in hair reflect the integrated hormone secretion of the past weeks or months, depending on the hair growth pattern and growth rate^6–8^. Thus, animal welfare may be improved and short-term fluctuations of endocrine secretion patterns are levelled out by hormone analysis in hair samples. The latter is of particular advantage for studying hormones in an ecological or evolutionary context.

In laboratory mice, Dry^9^ found in 1926 that one actively growing hair from the undercoat (85% of all hair follicles) is accompanied by two to three hairs from earlier hair cycles. Consequently, hair samples of younger individuals include fewer hair generations and reflect shorter time windows than samples from older individuals. In addition, the time of subadulthood (i.e. the first hair generation) is still represented in hair samples of younger adults, but probably not in older adults. A cycle of a single hair follicle roughly consists of 14 days of growth followed by 14 days at rest. The growth of neighbouring hair is highly synchronized. Therefore, events occurring during resting phases might not be represented at a particular location and each hair sample should reflect the past three to four months. Interestingly, a more recent shave and re-shave study on age controlled laboratory house mice found that hair had visibly regrown to 90–100% in 88 of 115 animals after nine weeks, but ranged from no regrowth to 75% regrowth in 27 mice^10^. This indicates significantly more individual variation in the hair growth patterns of mice than suggested by Dry^9^.

The glucocorticoid hormone corticosterone is the primary stress hormone in rodents, birds, fish and reptiles, and has been well studied because of its direct implications for health^11–13^. Physiological stressors (such as pain, hunger) or psychological stressors (such as social conflicts) activate a hormonal cascade of the hypothalamic-pituitary-adrenal (HPA) axis, which results in elevated corticosterone secretion into the blood stream. Corticosterone contributes to an increased energetic availability via gluconeogenesis and the metabolism of fat and proteins. As a trade-off, energetically expensive pathways not needed for immediate survival are suppressed or down-regulated^14^. Consequently, this stress response mechanism is adaptive in coping with acute stressors whereas the same mechanism becomes maladaptive if stressors are persistent^15^. Without sufficient time to recover, a prolonged stress response reduces fitness, e.g., through increased susceptibility to infectious diseases^16,17^, inhibition of embryo implantation^18,19^, or male infertility^20^. Thus, from an evolutionary perspective the chronic state of corticosterone secretion, as measured in hair, is likely to be more informative than the acute state.

Recently, a small number of rodent studies have shown the potential for analysis of corticosterone in hair samples^13,21–26^. For example, corticosterone concentrations in hair and blood serum were positively correlated in both control and stressed groups^26^. There is still some uncertainty about the exact incorporation mechanisms of hormones into the hair shaft^27,28^ but it is commonly assumed that hormones are mainly incorporated in the hair shaft during growth^8^ and that hair hormones reflect the cumulative hormone secretion during the time of hair formation.

Studies with repeated administration of adrenocorticotropic hormone, which increases production and release of cortisol (the primary glucocorticoid in bigger mammals), resulted in significantly elevated hair cortisol concentrations in chipmunks and lynxes^29,30^. There are only a few studies of sex hormones from mammalian hair^10^. Some studies found higher testosterone in males than in females^31,32^ or higher in rutting than non-rutting roe deer males^33^, whereas others did not find the expected difference^30,34–38^. Progesterone has been used successfully to distinguish between sexes^30,35^ or the reproductive state of females^35,39^. However, none of these studies has investigated rodents.

Here we use hair samples to evaluate long-term levels of corticosterone and sex steroid hormones from wild-derived house mice (*Mus musculus domesticus*) under conditions of reproductive competition in semi-natural enclosures. We expect that reproductive competition will enhance variation in hair hormone levels in predictable ways.

First, we predict that higher hair corticosterone will be associated with stress as possibly reflected in the number of visible injuries or scars. Louch and Higginbotham^40^ found that subordinate male mice were regularly victims of dominant males’ aggression and showed higher levels of corticosterone concentrations in blood serum than dominants. Subordinates also showed a high rate of wounds on the tail and rump. In comparison, dominant males received very few injuries^40^. In addition, males repeatedly exposed to aggressive confrontations showed significantly elevated plasma corticosterone concentrations^41^.

Second, if hair samples truly reflect longer-term endocrine secretion patterns, we predict that concentrations of the sex steroid hormone testosterone will be higher in males than females, and increase in males at sexual maturity. Testosterone is biologically active in both males and females, but exhibits higher concentrations and greater relevance in males. Predominantly synthesized in the testes, testosterone concentrations in males rise significantly during sexual maturation, where this hormone promotes the development of male reproductive organs and other secondary sexual traits. In addition, testosterone concentration is closely associated with sexual activity (for review see Vignozzi *et al*.^42^). We therefore predict positive correlations between testosterone and traits of male sexual maturity, like testes size^33,43^ or the number of sperm in the cauda epididymis, the location of final sperm maturation and sperm reservoir^44,45^. In females, smaller amounts of testosterone are synthesized in the ovaries and the adrenal cortex, but concentrations were found to be predictive for behavioural traits, such as suboptimal maternal breeding behaviour^46^.

Third, we predict that the steroid hormone progesterone will be found at higher concentrations in hair of females than in males, will increase in females at sexual maturity as well as in females that have been pregnant. Progesterone is one of the most important sex hormones in females, with increased production by the corpus luteum during the second half of the female cycle. The placenta synthesizes even higher concentrations, especially during the second half of pregnancy. In addition, progesterone concentration increases significantly with higher numbers of implanted embryos^47^ or with litter size^48^. We therefore predict progesterone concentration in females to increase with reproductive activity. Small amounts of progesterone are also synthesized in male testicles and adrenal cortex, where it is important in male sexual behaviour^49^.

## Methods

### Study population

We collected hair samples from house mice that were part of a study designed to measure dispersal propensity. These mice descended from a well-studied population of free-living wild house mice^50^. One hundred and nineteen laboratory-born, wild-derived house mice were placed in 14 enclosures of 7 m² with initial densities between 4 and 16 animals (50% female). The aim of the original study was to test for associations between traits, the social environment and the propensity to leave the enclosures via a tube leading to a water bath with a refuge cage with food, water, and hiding space on the opposite side of the water bath. The refuge cage was checked daily and any mice in it were removed from the experiment. For the present study, all mice removed from the refuge cage (n = 11) were euthanized and hair sampled.

The enclosures were fenced-in to impede predation by cats or birds of prey. They were exposed to ambient weather conditions, but were protected from rain and direct sunlight. The mice had *ad libitum* access to food (a 50:50 mix of rolled oats and commercial rodent food, Vita-Balance diet for guinea pigs and hamsters, Landi Schweiz AG, Dotzigen, Switzerland) and water at four sites in each enclosure. Four nest boxes as well as sticks, straw, plastic tubes and bricks provided shelter. Low, incomplete walls provided additional structure. A thick layer of mouse bedding (Lignocel Hygenic Animal Bedding, JRS GmbH & Co.KG, Rosenberg, Germany) covered the concrete floor of the enclosures. The enclosures were regularly inspected for the presence of litters. When pups were estimated to be 13 days old according to morphological traits^50^, an ear punch was taken from each pup for genetic analysis.

The enclosures were initiated between April and October 2017, ran for an average of 117 ± 31 (SD) days each and were then shut down by catching and euthanizing all mice. Immediately after euthanasia, we collected hair samples from all mice with sufficient hair (230 males, 252 females), collected an ear punch for genetic analyses, and documented body weight as well as injuries and scars. For the latter, we used the four categories “no wound or scar”, “one minor wound or scar” (one healed or recent bite wound on tail or rump, missing one digit or part of tail), “several minor wounds or scars”, and “major wounds” (serious bite wounds over a larger area). Microsatellite markers were amplified following Auclair *et al*.^51^ and the genotypes of pups (with known birth dates) and euthanized mice were matched, in order to provide age estimates (±2 d) for subadult and adult mice born within the enclosures. In addition, we dissected 113 females (age range = 25–217 d) in order to count placental scars and developing embryos in the uterus, which inform about the total number of embryo implantations throughout life^52,53^. We also dissected 108 males (age range = 23–218 d) in order to measure testes weight (right and left testis combined; ±0.1 mg) and count sperm numbers in the cauda epididymis. To count sperm, we transferred both cauda epididymes to 1 ml of oil-immersed modified human tubal fluid (mHTF) medium (Catalog ID: 90126, FUJIFILM Irvine Scientific, Santa Ana, USA) supplemented with 5 mg/ml of bovine serum albumin (Sigma-Aldrich), pre-heated to 37 °C. We used three cuts on each epididymis to allow sperm to escape into the medium over 2 h in an incubator set at 37 °C. We then transferred a 4 µl sample from the centre of the medium bubble to a pre-warmed 20 micron Leja4 slide, and examined it on the heated stage of an Olympus CX41 phase-contrast microscope using 40X magnification. We used a CASA, computer assisted sperm analysis system (Mouse Traxx, Hamilton Thorne, Beverly, USA), to image sperm and estimate sperm concentration. We observed at least 200 sperm per slide, using multiple viewing panels. If the CASA system determined that sperm concentration was too high, we diluted samples 1:1 with pre-warmed mHTF medium^54^.

### Hair sample preparation and hormone analysis

Hair samples were shaved from the lower back of the animals with an electric razor after euthanasia (~15–20 mg hair, equivalent to a square of 2 × 1 cm), following a previous publication on laboratory mice^23^. Samples were kept in individual paper envelopes at room temperature until analysis. Hair sample preparation followed the protocol as described for human hair^55^. Slight adaptations were necessary in order not to lose the very short and fine mouse hair (~5 mm) during washing. Hair was washed once by shaking it in 1.5 ml of isopropanol for 3 min at room temperature after which the liquid was pipetted off. Hair was then allowed to dry for at least 12 h at room temperature. 5 ± 0.5 mg of non-pulverized hair was transferred into a 3 ml glass tube. Thereafter, 1.8 ml methanol was added and the hair was incubated for 18 h at room temperature for steroid extraction. 1.6 ml of the clear supernatant was transferred into a new 2 ml plastic tube (Eppendorf, Hamburg, Germany). The alcohol was evaporated at 50 °C under a constant stream of nitrogen until the samples were completely dried (duration: approximately 40 min). The dry residue was suspended using 225 μl distilled water (LC-MS-grade) and 25 μl methanol. 100 μl of the re-suspension, together with 20 μl of an internal standard were used for liquid chromatography - tandem mass spectrometry (LC–MS/MS) analysis. The LC–MS/MS analysis followed our previously validated protocol for corticosterone, testosterone and progesterone^55^. We had intended to also measure estradiol in the hair samples, however, concentrations were below the detection level of the LC–MS/MS method used here.

### Statistical analyses

All statistical analyses and figures were performed in R 3.4.4^56^ with *RStudio*^57^ and the packages *ggplot2* 3.0.0^58^ and *lme4* 1.1–18.1^59^, the latter using the functions *lmer and confint.merMod*.

Hormone measures below the limit of detection of 0.1 pg/mg hair^55^ were excluded from further analyses (n(testosterone) = 54; n(progesterone) = 14). Further exclusion criteria included missing records on injuries/scars (n = 1) and unknown age (n = 43).

We used linear mixed-effects models in a model building approach to test the effect of sex and age, their potential interaction, and body weight on the three investigated hormones. We included the identity of each enclosure as a random effect in our models. Data for all three hair hormones were not normally distributed, but approached normal distribution with a log transformation. In order to improve readability, we back-transformed the logarithmic beta-values from these models and calculated 95% confidence intervals.

We investigated whether the 95% confidence intervals of the testosterone and progesterone model estimates would allow us to determine at which age males and females diverged from each other hormonally. This age was interpreted as the beginning of adulthood visible in hair hormones, with animals younger than this separation age defined as subadults.

The level of injuries and scars in mice were used to infer the severity of social conflicts or received aggression an animal had to cope with. To test for an association between injuries and corticosterone concentrations, we compared the fit of models that included or excluded injury level.

In females, we assessed the relationship between hair hormone levels and markers of pregnancy, namely the number of embryo implantations and the number of litters produced during the experiment. In males, we assessed the relationship between testosterone concentrations and testes weight as well as number of sperm in the cauda epididymis as a measure for sexual maturity. 4^th^ root transformation was applied to sperm number in order to reduce skew in this measure.

Body weight was available only at the time of euthanasia, differed between the sexes (with males heavier than females; Welch two sample t-test, t = 4.32, df = 482.7, p < 0.0001), and was highly correlated with age at sampling (r = 0.85). Consequently, the predictors in our models showed increased collinearity (variance inflation factors between 3 and 5), suggesting reduced potential for significant results. Despite this, our predictor variables kept their strong explanatory power in models where body weight was included. We therefore decided to keep body weight in our models.

## Results

**Figure 1:**
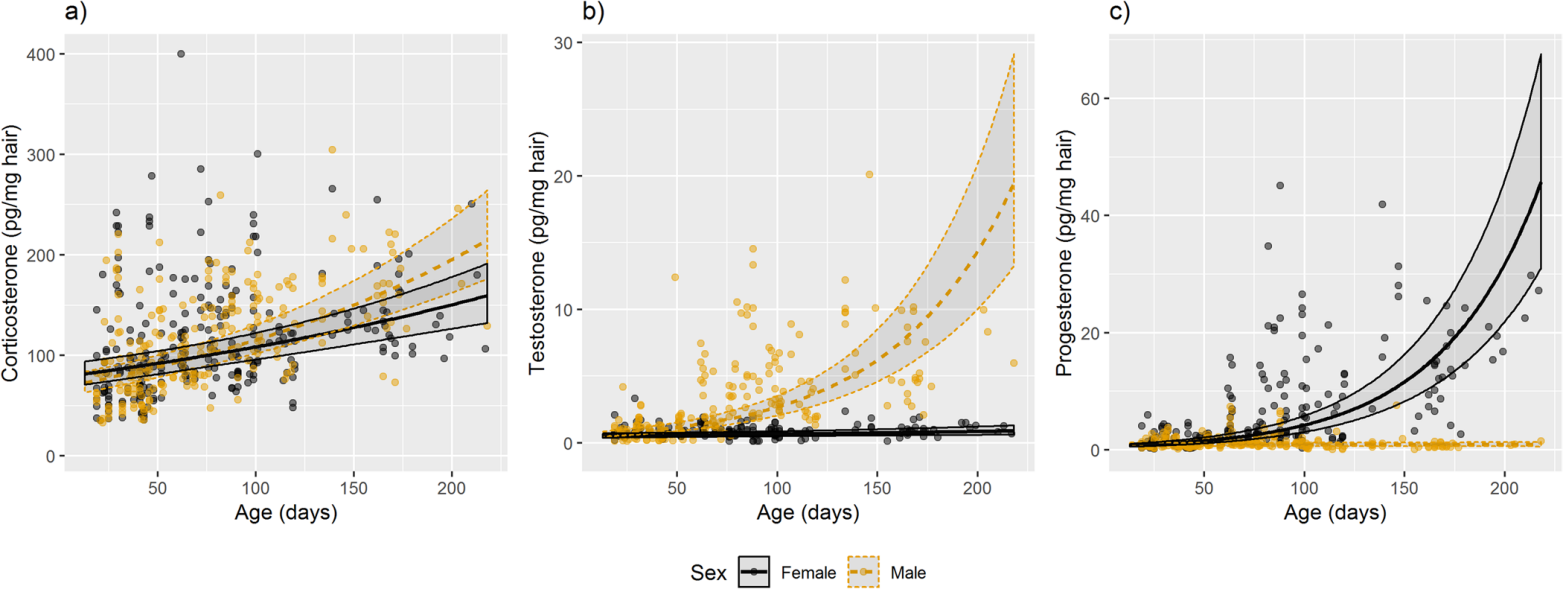
The influence of age and sex on three hair steroid hormones. Scatter plots illustrating the effects of sex and age at sampling on (**a**) corticosterone, (**b**) testosterone, and (**c**) progesterone measured in hair of wild-derived house mice. Data (dots and lines) for females are given in grey, for males in ochre.

An interaction between age at sampling and sex predicted levels of the three steroid hormones corticosterone, testosterone and progesterone measured in hair of wild-derived house mice (Fig. 1, Table 1). For corticosterone (Fig. 1a), age had a strong positive effect (corticosterone Model 2 *vs*. Null model, Table 1), while sex itself did not have explanatory power (corticosterone Model 1 *vs*. Null model, Table 1). However, fitting an interaction of age and sex showed that older males had higher corticosterone levels than older females (corticosterone Model 4 *vs*. Model 3, Table 1 and Fig. 1a). Adding body weight to the model did not yield better model fit (corticosterone Model 5 *vs*. Model 4, Table 1).

**Table 1:** Overview of the model comparisons for the prediction of hair corticosterone, hair testosterone, and hair progesterone in house mice. The ‘x’ indicates model term interaction.

Model	Formula	comparison	*Χ*²	*p*-value	ΔAIC
Null model with random effect	**log(corticosterone)** ~ (1|Enclosure)				
Model 1	~sex	Null model	0.00	0.95	+2.0
Model 2	~age	Null model	97.0	<0.001	−95.0
Model 3	~sex + age	Model 2	0.2	0.66	+1.8
Model 4	~sex × age	Model 3	7.3	<0.01	−5.3
Model 5	~sex × age + body weight	Model 4	0.2	0.64	+1.78
Model 6	~injury	Null model	78.3	<0.001	−76.3
Model 7	~sex × age + injury	Model 4	23.5	<0.001	−21.5
		Model 6	49.6	<0.001	−43.7
Null model with random effect	**log(testosterone)** ~(1|Enclosure)				
Model 1	~sex	Null model	125.2	<0.001	−123.2
Model 2	~age	Null model	55.1	<0.001	−53.2
Model 3	~sex + age	Model 1	105.8	<0.001	−103.8
Model 4	~sex × age	Model 3	87.9	<0.001	−85.9
Model 5	~sex × age + body weight	Model 4	30.9	<0.001	−28.8
Null model with random effect	**log(progesterone)** ~(1|Enclosure)				
Model 1	~sex	Null model	155.0	<0.001	−153.0
Model 2	~age	Null model	126.6	<0.001	−124.6
Model 3	~sex + age	Model 1	165.3	<0.001	−163.3
Model 4	~sex × age	Model 3	210.6	<0.001	−208.6
Model 5	~ sex × age + body weight	Model 4	20.4	<0.001	−18.4
Null model with random effect	**log(progesterone) females only** ~ (1|Enclosure)				
Model 1	~ age	Null model	110.9	<0.001	−108.9
Model 2	~ body weight	Null model	89.6	<0.001	−87.6
Model 3	~ age + body weight	Model 1	4.2	<0.05	−2.2
Model 4	~ age + body weight + no. litters	Model 3	1.1	0.29	−0.9
Model 5	~ age + body weight + no. implantations	Model 3	13.5	<0.001	−11.5
Null model with random effect	**log(testosterone) males only** ~ (1|Enclosure)				
Model 1	~ age	Null model	79.1	<0.001	−77.1
Model 2	~ body weight	Null model	96.6	<0.001	−94.6
Model 3	~ body weight + age	Model 2	8.0	<0.01	−6.0
Model 4	~ body weight + age + testes weight	Model 3	23.1	<0.001	−21.1
Model 5	~ body weight + age + (sperm number)^1/4^	Model 3	18.1	<0.001	−16.1
Model 6	~ body weight + age + testes weight + (sperm number)^1/4^	Model 4	4.5	<0.05	−2.5

**Figure 2:**
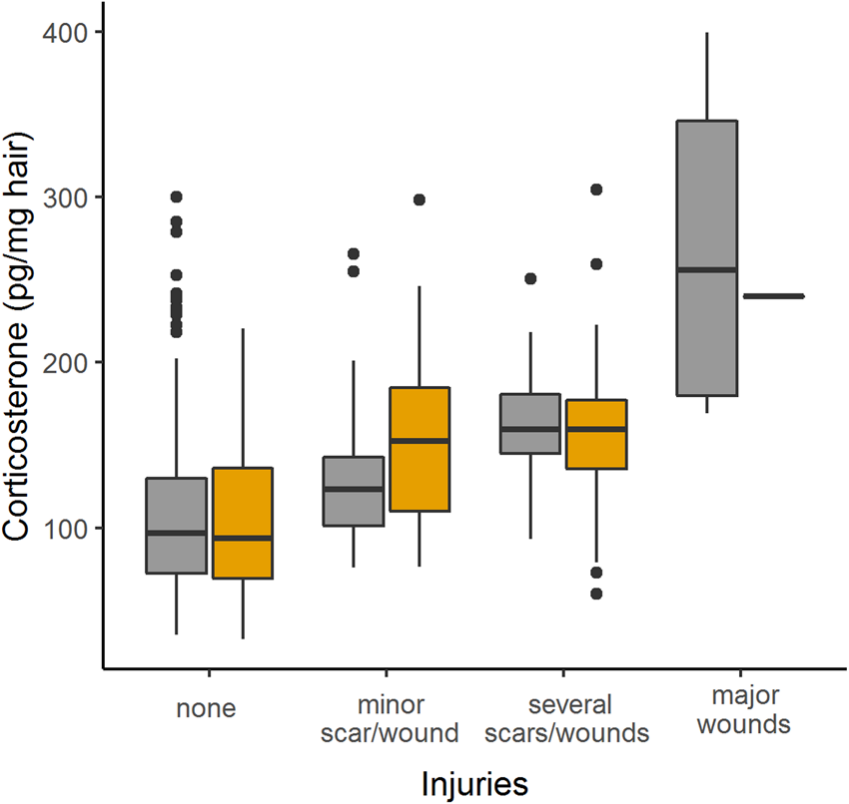
The impact of the level of injury on hair corticosterone. Box and whisker plots (with median, 1^st^ and 3^rd^ quartile and outliers) illustrating the level of injuries in relation to corticosterone measured in hair from wild-derived female (in grey) and male (in ochre) house mice. See Methods for injury categories.

The level of injury had a strong predictive effect on corticosterone concentration which was additive to the effects of sex and age (corticosterone Model 7 *vs*. Model 4 and Model 7 *vs*. Model 6, Table 1, Fig. 2). Using the back-transformed estimate of the sex and age interaction term in model 7 of 1.0010 (95% CI = 0.9994–1.0026), we calculated that with each additional month of age corticosterone increased 3% more in males compared to females. The back-transformed estimate of effect of injury of 1.1539 (95% CI = 1.0910–1.2206), predicts an increase of corticosterone by 15% with each level of injury, independent of the effects of age and sex. Injuries of the highest category (“major wounds”) occurred on the animals’ back. As a consequence, mice with major wounds had little hair on their back and thus were rarely sampled (hair samples could only be collected from 1 male and 4 females). Nevertheless, more males than females were sampled in the second highest category “with several scars/wounds”, suggesting that fewer females suffered from injuries in this category than males (injury distribution females/males: “none” = 217/172; “one minor wound/injury” = 22/19; “several wounds/injuries” = 13/41; “major wound” = 4/1).

The concentration of testosterone in hair (Fig. 1b) differed significantly between males and females (testosterone Model 1 *vs*. the Null model, Table 1) and increased with age (testosterone Model 2 *vs*. the Null model, Table 1). The interaction of age and sex was significant (testosterone Model 4 *vs*. Model 3, Table 1), indicating that the slopes of change of testosterone concentrations differed between the sexes. Adding body weight at the time of euthanasia to this model further improved our model fit (testosterone Model 5 *vs*. Model 4, Table 1). Thus, older males exhibited significantly higher testosterone than older females (back-transformed estimate = 1.0141, 95% CI = 1.0115–1.0167). Testosterone in males increased per month of age 43% more in males than in females and increased by 7% by each gram of body weight (back-transformed estimate = 1.0676, 95% CI = 1.0424–1.0918). The confidence intervals of testosterone values stopped overlapping in males and females at the age of 38 days. Males younger than 38 days were therefore categorized as subadults, otherwise as adults.

Hair progesterone concentrations (Fig. 1c) also differed between the sexes (progesterone Model 1 *vs*. Null model, Table 1), with females showing considerably higher levels. Age positively influenced progesterone levels (progesterone Model 2 *vs*. Null model, Table 1), as did the interaction between sex and age (progesterone Model 4 *vs*. Model 3, Table 1). Similar to testosterone, progesterone was furthermore significantly predicted by body weight at the time of euthanasia (progesterone Model 5 *vs*. Model 4, Table 1). Using the estimated slope value for the difference in effect between males and females of 0.9801 (95% CI = 0.9778–0.9826), progesterone concentrations increased per month of age 60% more in females than in males and there was also a 4.6% increase of progesterone with each gram of body weight (back-transformed estimate = 1.0465, 95% CI = 1.0255–1.0672). The progesterone model showed that confidence intervals of the two sexes no longer overlapped at the age of 54 days. Thus, females younger than 54 days were categorized as subadults, otherwise as adults.

**Figure 3:**
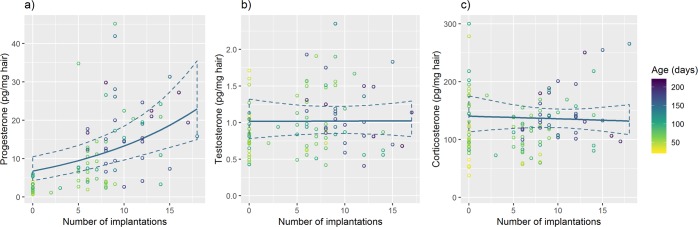
The impact of the number of implantations on three hair steroid hormones. Scatter plots of the number of embryo implantations against progesterone (**a**), testosterone (**b**) and corticosterone concentration (**c**) in hair of wild-derived female house mice, as a function of age. The illustrated line shows the model prediction (with 95% confidence intervals) for females at 150 days of age.

For females, adding the number of embryo implantations throughout life improved the fit of the model containing age and body weight (progesterone Model 5 females only *vs*. Model 3, Table 1). Using the back-transformed slope estimates for age of 1.0091 (95% CI = 1.0042–1.0136), for body weight of 1.0289 (95% CI = 0.990–1.0725), and for number of embryo implantations of 1.0659 (95% CI = 1.0301–1.10; Fig. 3a), among females, progesterone was estimated to increase by 27.2% per month of age, by 3% per gram of body weight, and by 6.6% with each implanted embryo. The number of litters produced by a female (mean = 0.9, range = 0–4) had itself explanatory value against the Null model (data not shown) but did not add explanatory value compared to a model that contained age and body weight (progesterone Model 4 females only *vs*. Model 3, Table 1). A significant correlation with medium effect size between progesterone and age was still detectable among those 38 females without any signs of pregnancy (r = 0.39, p = 0.02; age range = 40–140 d). Otherwise, the number of implantations did not significantly correlate with female testosterone (Fig. 3b) or corticosterone (Fig. 3c), neither in young nor in older females.

**Figure 4:**
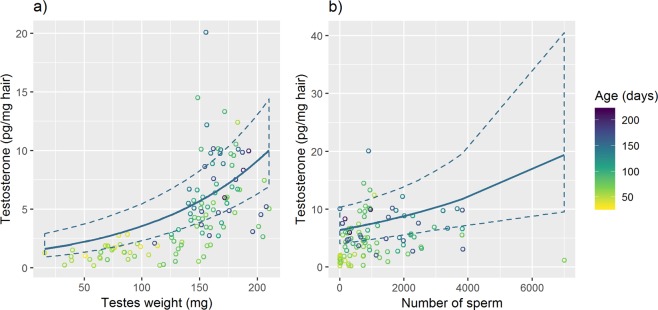
The impact of testes weight and sperm number on testosterone. Scatter plots of testes weight (**a**) and number of sperm (**b**) against testosterone in hair of wild-derived male house mice, as a function of age. The illustrated line shows the model prediction (with 95% confidence intervals) for males at 150 days of age.

Testosterone in males was significantly predicted by body weight (strongest predictor), age and testes weight (testosterone Model 4 males only *vs*. Model 3, Fig. 4a). The number of sperm additionally improved the model fit (testosterone Model 5 males only *vs*. Model 3, Fig. 4b). The back-transformed slope estimates for testes weight of 1.0064 (95% CI = 1.0025–1.0106) and for sperm number of 1.000013 (95% CI = 1.0–1.00017) indicate a testosterone increase by 0.6% per mg increased testes weight (mean testes weight = 134 mg, range = 13–221 mg) and 1.3% increase for each 1000 sperm counted (mean sperm count = 998, range = 0–7000). Testosterone further increased by 20% per month of age (back-transformed slope estimate = 1.0066, 95% CI = 1.0030–1.0100) and by 4% per increased gram of body weight (back-transformed slope estimate = 1.0396, 95% CI = 0.9931–1.0830).

## Discussion

This is the first demonstration of cumulative steroid hormone analysis in hair samples from a larger number of wild-derived house mice, living in social groups consisting of multiple males and females exposed to intra-sexual reproductive competition. Hair corticosterone analysis has previously been validated and used for rodents under laboratory conditions, but not under field conditions. In addition, biological validation for hair testosterone and progesterone has still been pending. As predicted, we found that sex and age of sampling, either alone or in interaction, influenced concentrations of corticosterone, testosterone and progesterone. We used these patterns to determine the age of sampling at which hair hormone concentrations of testosterone and progesterone diverged between the sexes, reflecting the onset of adulthood (sexual maturation) and potential intra-sexual reproductive competition. Progesterone and testosterone concentration, but not corticosterone, further increased with body weight at the time of euthanasia. In males, testosterone was more strongly predicted by body weight than by age. We also found that higher corticosterone concentrations were associated with higher levels of injury, suggesting chronic social stress in some animals. Furthermore, the total number of implanted embryos positively predicted progesterone levels, as expected. Unexpectedly, we found similarly high levels of corticosterone in females and in males, which also increased with age at sampling. We first discuss the time window over which hormones are incorporated in the hair sampled, and then use the full set of results from this method to test predictions about intra-sexual reproductive competition.

Our study provides indirect information on the time window represented in wild mouse hair samples. Both, the significant positive associations between hair progesterone and the number of embryo implantations throughout a female’s life (maximum 6 months) as well as between corticosterone and the number of scars and injuries throughout life suggest that hair samples from house mice are an integrated measure of a broad time window of several months. Nevertheless, the question about the exact time window that is represented in a hair sample of wild house mice cannot be answered here.

Looking at the sex steroid hormones in hair as a correlate of reproductive behaviour, we found strongly elevated testosterone in older males and, analogously, largely increased progesterone in older females, which is expected given the nature of testosterone and progesterone. These results provide evidence for the biological validity of measurements of the two sex steroids in hair in house mouse hair. This is important as a recent review article^10^ emphasized the need for further validation of reproductive hormones in hair. Surprisingly, there are more studies that did not find higher testosterone concentrations in males than females^30,34–38^, than those that did^31,32^.

Similarly to a study on brown bears^60^, where sex hormone profiles enabled the categorization of age classes, our models on testosterone and progesterone allowed us to determine a threshold age at which male and female wild-derived house mice began to differentiate in their hormone concentrations from the opposite sex. We interpret this age as the onset of adulthood (sexual maturation) that is reflected in hair hormone measures. We would expect to see a first increase in sex hormones even earlier since cumulative hormone levels measured in hair provide a retrospective measure over several weeks rather than acute hormone levels. Nevertheless, given that the hormone concentrations measured in hair accumulated over weeks or even longer, our hair hormone-based age of adulthood in wild-derived males (38 d) and females (54 d) is surprisingly close to the reported onset of reproduction in male (28–34 d) and female laboratory mice (first oestrus in females: 35–42 days^61^). In the free-living population from which the study mice descended, onset of first reproduction varies drastically among individuals. The earliest age when males sired their first litter was 31–35 days of age, and females gave birth to their first litter the earliest when 51–58 days old (unpublished data).

Looking at progesterone in females specifically, we found that the number of embryo implantations throughout life, based on the number of scars and embryos found in the uterus, was a much stronger predictor of progesterone than the number of litters produced by a female. In fact, the number of litters did not explain further variation in progesterone if age was included in the model. This is not surprising given that the number of litters observed in this study might not correspond to the number of pregnancies, due to loss of litters through abortion or infanticide before weaning^51,62,63^. More than one third of the adult females (41 of 113) showed signs of embryo implantations although we never found litters from these females. To our knowledge, this is the first time that progesterone has been found to be more strongly linked to number of implantations than number of litters produced.

Testosterone in male mice was most strongly predicted by body weight, which is in line with the hormone’s key role in muscle development and fat deposition in males^64,65^. Interestingly, age, in addition to body weight, was also a significant predictor for male testosterone. This was unexpected, considering the high correlation between age and body weight (r = 0.81), as house mice increase in size throughout life^66^. Importantly, testes size and (to a lesser degree) sperm number in the cauda epididymes, indicators of sperm production capacity^67^ and reproductive activity^44^, respectively, were further significant predictors for male testosterone. Similar associations between testes size and plasma testosterone have been reported in other animals^68,69^. All these findings highlight that testosterone concentration measurements in the hair of male mice reflect male sexual maturity. They support the biological validity of hair testosterone measurements, along with findings on hypogonadal men^70^ and rutting roe deer^33^.

Concerning stress hormones and injuries as proxies of reproductive competition, our results indicated that corticosterone concentrations measured in hair were remarkably similar between males and females, elevated only in older males compared to older females. This is surprising, as in laboratory settings more direct aggressive interactions between males than between females occur^71^, reflected in typical housing conditions, as in many strains, females, but not males, can be housed together^72^. Although more prevalent among males, injuries were not absent from females. Female injuries arise from aggression by males or females, as during territorial aggressive interactions^71,73^. In house mice, sexual coercion has not been observed^74,75^ despite sexual size dimorphism^66,76^. Recent ideas of the regular occurrence of reproductive competition between females^3,77–79^ suggest that group living females compete with each other over resources they need to reproduce. This idea is supported by the 38 adult females in this study that showed no signs of pregnancy, or scars left behind by previous implantations of embryos in the uterus. Given that food had not been limited and was easily accessible in our study, females might have rather competed over access to safe nest sites, or protection or assistance with offspring care. Such intrasexual competition in female house mice need not necessarily take the form of direct aggression and visible injuries, but rather indirectly suppress the competitors’ reproduction through olfactory signals that communicate social dominance (for review see Stockley and Bro-Jørgensen^3^), or through infanticide towards their competitors’ offspring^62,80,81^. Prolonged stress associated with social harassment can further have long-term consequences for reproductive success through reduced lifespan^82,83^. Stockley and Bro-Jørgensen in their recent review^3^ emphasised that female adaptations for intrasexual competition are often less conspicuous than those of males. As a consequence, they are generally more challenging to study. Analysing stress hormones in hair may fill that gap and reveal a stressful social environment even in the absence of overt aggression.

In summary, we found strong support for the biological validity of the stress hormone corticosterone and the sex steroid hormones progesterone and testosterone as measured in the hair of wild-derived house mice. Although the exact time window needs further investigation, the present study supports the notion that hormone measures from the hair of wild house mice reflect several weeks of hormone accumulation. In comparison, most hormone measures derived from blood, urine, or faeces, rather reflect the acute endocrine state. We feel confident that the broader time window from hair hormone measures will open new avenues to study the role of the social environment on reproductive competition in wild populations. Bronsen^84^ noted in 1989, which holds true today: “Also important, but largely missing, are good hormonal data obtained from wild populations. How does one collect such data given the episodic nature of secretion of many of these hormones and the interfering stress of capture? Some truly imaginative thinking will be required to solve this problem.” Hair hormone analysis is likely able to fill that gap in many species.

### Ethics

The data were collected under permit ZH134/16 from the Cantonal Veterinary Office.

## Data Availability

The dataset supporting this article is deposited in Dryad: 10.5061/dryad.x95x69pd6.
